# Effects of IL-1β and TNF-α on the Expression of P311 in Vascular Endothelial Cells and Wound Healing in Mice

**DOI:** 10.3389/fphys.2020.545008

**Published:** 2020-11-27

**Authors:** Daijun Zhou, Tengfei Liu, Song Wang, Weifeng He, Wei Qian, Gaoxing Luo

**Affiliations:** ^1^Institute of Burn Research, State Key Laboratory of Trauma, Burn and Combined Injury, Southwest Hospital, Army Medical University, Chongqing, China; ^2^Department of Oncology, General Hospital of Western Theater Command of PLA, Chengdu, China

**Keywords:** wound healing, vascular endothelial cells, P311, TNF-α, IL-1β

## Abstract

**Objective:**

This study aimed to define the role of interleukine-1β (IL-1β) and tumor necrosis factor-α (TNF-α) in the expression of P311 in vascular endothelial cells (VECs) and in wound healing.

**Methods:**

DAPI staining, a CCK-8 assay, cell migration assay, and an angiogenesis assay were used to assess the effects exerted by TNF-α and IL-1β at various concentrations on morphology, proliferation, migration, and angiogenesis of VECs. Western blot (WB) and reverse transcription-polymerase chain reaction (RT-PCR) models were employed to observe the effects exerted by proteins related to the nuclear factor-kappa B (NF-κB) signaling pathway and P311 mRNA expression. Bioinformatics analysis was performed on the binding sites of P311 and NF-κB. Finally, to investigate the effects of IL-1β and TNF-α on wound healing and the length of new epithelium in mice, we established a full-thickness wound defect model in mice. Immunohistochemistry was used to measure changes in P311, proliferating cell nuclear antigen (PCNA), vascular endothelial growth factor (VEGF), CD31 (platelet endothelial cell adhesion molecule-1, PECAM-1/CD31), as well as other related proteins.

**Results:**

When levels of TNF-α and IL-1β were both 20 ng/ml, their effects on cell proliferation, cytoskeleton protein expression, cell migration, and angiogenesis were the greatest (*P* < 0.05). IL-1β and TNF-α at moderate concentrations effectively promoted P311 mRNA and p-NF-κB protein expression (*P* < 0.05), while p-NF-K b protein expression was decreased (*P* < 0.05). Luciferase assays showed that P311 expression was also relatively greater when stimulated at moderate concentrations (*P* < 0.05), while relative expression was significantly lower when the p-NF-K b inhibitor CAPE was added (*P* < 0.05). On 7-day wound healing rate comparison, the control, IL-1β, IL-1βab, TNF-α, and TNF-αab groups were 18, 37, 35, 39, and 36%, respectively, while control group + P311 siRNA was 31% (*P* < 0.05). New epithelial length, granulation tissue thickness, and number of blood vessels trends were also the same. In the control group, P311 showed lower relative expression levels than the others (*P* < 0.05). P311 relative expression levels trended as follows: control group > IL-1βab > IL-1β > TNF-αab > TNF-α (*P* < 0.05).

**Conclusion:**

When IL-1β and TNF-α concentrations are moderate, they effectively promote the proliferation, expression, migration, and angiogenesis of VECs, possibly by promoting the expression of the NF-K b pathway and thereby promoting the expression of P311. *In vitro* experiments on mice suggest that P311 effectively promotes wound healing, and its mechanism may be closely related to PCNA, CD31, and VEGF.

## Introduction

P311, also known as NERP, refers to a highly conserved 8-kDa intracellular protein (Branco et al., 2014). In the N-terminus, P311’s 68-amino acid sequence includes a PEST domain (rich in Pro, Glu, Ser, and Thr). The domain is also found in short-lived ephemeral proteins such as transcription factors, cytokines, and signal molecules, suggesting that P311 might belong to one of these protein families (Brun et al., 2003; Cheng et al., 2017).

Angiogenesis, the process by which new blood vessels are established from pre-existing ones, is critical to wound healing. Microvascular endothelial cells are the principal parenchymal cells participating in wound angiogenesis (Curreri et al., 1975; Eming et al., 2007) that involves a phenotypic alteration of endothelial cells, directed migration, and various mitogenic stimuli. Our previous studies demonstrated that P311 was a crucial factor in wound healing. However, it remains unclear as to how P311 is regulated in wounded endothelial cells so as to exert its biological function (Curreri et al., 1975). In the early stage of wound healing, activated platelets, neutrophils, macrophages, and other bioactive mediators are produced due to stress, inflammation, and hypoxia. In particular, interleukine-1β (IL-1β), tumor necrosis factor-α (TNF-α), and persistent hypoxia stimulation are key factors involved in the initiation of wound healing (Curreri et al., 1975; Eming et al., 2007).

The high levels of expression of P311 in wounds suggest that inflammatory factors and hypoxia and the microenvironment of the wound may be key factors in activating the expression of P311. In our preliminary experiments, we found that TNF-α and IL-1β increased P311 mRNA expression in vascular endothelial cells (VECs) by approximately 50-fold, while IL-6 and hypoxia increased levels of P311 mRNA in VECs by approximately 10-fold (Aittokallio and Schwikowski, 2006; Badri et al., 2013). Therefore, we speculated that inflammatory stimulation and hypoxia after burns and other microenvironmental factors may be factors causing the increased expression of P311 (Becker et al., 2011; Bolliger et al., 2012). The present study aimed to define the role of IL-1β and TNF-α in the expression of P311 in VECs as well as in the process of wound healing.

## Materials and Methods

### *In vitro* Inflammatory Stimulation Model

To investigate the response of VECs to inflammatory stimuli, we used human umbilical vein endothelial cells (HUVEC) to construct an *in vitro* inflammatory stimulation model. Briefly, HUVEC underwent stimulation with IL-1β or TNF-α in various concentrations for 24 h. TNF-α or IL-1β at 4, 20, and 100 ng/ml were defined as the groups with severe stimulation, moderate stimulation, and mild stimulation, respectively. HUVEC without stimulus factors were used as the control. After 24 h of stimulation, further experiments were performed to analyze VEC responses to inflammatory stimulus.

### Cell Cytoskeleton Fluorescent Staining Assay

To study the influence of inflammatory stimulation on VEC cytoskeleton morphology, HUVEC were inoculated on coverslips for 24 h in media with various concentrations of IL-1β or TNF-α as mentioned above. Then, cells were washed three times with preheated phosphate-buffered saline (PBS) and then were fixed in 4% paraformaldehyde preheated at room temperature. We then washed and immersed the fixed cells in a PBS that contained 0.5% Triton X-100 for 5 min at room temperature. The cells were then stored with TRITC-coupled phalloidin prepared for 30 min at 37°C without light, and DAPI for 5 min. Fluorescent images of the stained cells were obtained under a confocal laser scanning microscope (780, Zeiss, Germany).

To determine whether inflammatory stimuli affected VEC cytoskeleton morphology by regulating the expression of P311, P311 siRNA was used to downregulate the expression of P311. We introduced P311 siRNA into a HUVEC culture medium with various concentrations of IL-1β or TNF-α, followed by a 24-h incubation. Then the HUVEC were stained with DAPI and phalloidin before fluorescent observation.

### Cell Proliferation Assay

To measure the impact of inflammatory stimulation on VEC proliferation, HUVEC were seeded in 96-well plates and stimulated with various concentrations of TNF-α or IL-1β. After stimulation for 24 h, we disposed of the culture medium. Then, we added 100 μl of fresh medium and 10 μl of CCK-8 reagent (Dojindo, Kyushu, Japan) to every well. The cells underwent incubation at 37°C for 4 h, and then were measured using an enzyme-linked immunosorbent assay microplate reader (Thermo Varioskan Flash, United States). The sample absorbance at 450 nm represented the number of living cells.

To determine whether inflammatory stimuli influenced VEC proliferation by regulating the expression of P311, we introduced P311 siRNA into the culture medium of HUVEC with various concentrations of TNF-α or IL-1β for a 24-h incubation, followed by the detection of cell proliferation using CCK-8 assays.

### Cell Migration Assay

A scratch wound migration assay was used to determine the migration ability of VECs after inflammatory stimulation, as previously reported. Briefly, HUVEC were seeded into 24-well plates and cultured to nearly 90% confluence. Mitomycin C (Sigma, United States, M4287) at 4 ng/ml was used to treat cells for 2 h to suppress cell proliferation. Then, a cell-free vertical scratch in the monolayer (0 h) was made at the center of the plate bottom using a sterile 200 μl plastic pipette tip. The culture medium and detached cells were removed. The cells were then washed three times. We subsequently added TNF-α or IL-1β at various concentrations in the clean culture medium. Cells were observed and photographed using a Zeiss video microscope at 0, 12, and 24 h after scratching. The remaining area was measured using the Image J software. The rates of cell migration were obtained as follows: cell migration rate = (initial area–remaining area at each observation point)/initial area × 100%.

To determine whether inflammatory stimuli would affect VEC migration through P311, we introduced P311 siRNA into the HUVEC culture medium with various concentrations of TNF-α or IL-1β for a 24-h incubation, followed by the detection of cell migration rates with the scratch wound migration assay.

### *In vitro* Tube Formation Assay

We conducted tube formation assays according to a previous report. In brief, 2 × 10^4^ HUVEC were seeded into Matrigel-coated 96-well plates, followed by the addition IL-1β or TNF-α at various concentrations. After 24 h stimulation, the newly formed tubes were photographed using an inverted phase microscope. To determine whether inflammatory stimuli affected VEC angiogenesis through P311, we introduced P311 siRNA into the culture medium of HUVEC with various concentrations of TNF-α or IL-1β for a 24-h incubation before the tube formation assay. The length of tubes and the number of tubes were measured using the Image J software. Each group contains six replicates. Each experiment was performed three times.

### Western Blotting Analysis

HUVEC at 2 × 10^6^/mL concentration were seeded into six-well plates containing the culture medium, followed by the addition of TNF-α or IL-1β at various concentrations. After 24 h stimulation, cells were lyzed in RIPA buffer to quantify total cell proteins. Concentrations of protein in the cell lysate supernatants were determined using a bicinchoninic acid (BCA) protein assay kit (Thermo Scientific, Rockford, United States). Then, we separated extracted proteins on 10% SDS-PAGE gels and transferred them to polyvinylidene difluoride (PVDF) membranes (Millipore Immobilon, United States). After being blocked with 3% BSA (Biosharp, CN, United States) for 2 h at room temperature with primary antibodies and the horseradish peroxidase (HRP)-conjugated secondary antibodies, nuclear factor-kappa B (NF-κB) and phospho-NF-κB expression were measured using western blotting (WB). β-Actin protein served as the internal reference. Using an enhanced chemiluminescence (ECL) detection kit (Pierce, 35055) and the ChemiDoc^TM^ XRS Imaging system (Bio-Rad, United States), we quantified chemiluminescence bands.

To determine whether inflammatory stimuli affected levels of phospho-NF-κB and NF-κB in VECs through P311, we introduced P311 siRNA into the HUVEC culture medium with various concentrations of TNF-α or IL-1β for a 24-h incubation before measuring protein expression of phospho-NF-κB and NF-κB using WB.

### Quantitative Real-Time PCR

After being incubated with different concentrations of IL-1β or TNF-α with/without adding CAPE for 24 h, we lyzed the cells and acquired total RNA using the RNeasy Mini Kit (QIAGEN, 74104). Using a DU800 UV/Vis spectrophotometer (Beckman Coulter, United States), we measured RNA concentration and quality. In accordance with the instructions of the manufacturer, mRNA underwent reverse transcription using a cDNA Synthesis Kit (TOYOBO, FSK-100). We performed real-time PCR using the 7500 Real Time PCR System (Applied Biosystems Instruments) with the following primers: 5′-AAGTGGAGGTAACTGATTCT TGG3′, 5′-GAGGCTTCCTAAGGGAAGACTT-3′ and P311: 5′- AGTGGGAGTTGCTGTTGAAGTC-3′, 5′CGTGCCGCCTGGA GAAAC-3′, and GAPDH, using SYBR Green Master Mix (TOYOBO, QPK-201).

### Effect of NF-κB on the Activity of P311 Promoter in Vascular Endothelial Cells and Analysis of Binding Sites

The transcription factor binding site of NF-κB to regulate P311 expression was predicted using the bioinformatics method. The sequence of the P311 promoter was obtained from the NCBI Gene database. The possible binding sites of NF-κB to the P311 promoter were then predicted by searching the JASPAR database.

To determine the effect of NF-κB on the activity of the P311 promoter, an NF-κB adenovirus and P311 promoter region luciferase reporter gene vector were constructed. Briefly, PCR amplification of the P311 promoter sequence was performed using the KOD-plus high-fidelity enzyme. The pGL3 basic vector and the TA cloning vector (PTA-P311) were digested with *Kpn*I and *Nhe*I, and the digested product was recovered. T4 ligase was used to ligate the digested product, and the plasmid was transformed and extracted. The two newly recombined vectors were subjected to the *Kpn*I and *Nhe*I double enzyme digestion system for identification, and the P311 promoter plasmid and NF-κB were transfected into HUVEC cells. After incubation with various concentrations of TNF-α or IL-1β with/without adding CAPE for 24 h, cells were lyzed and P311 promoter activity was measured using a luciferase activity.

### *In vivo* Wound Healing Assay

We established a murine-infected full-thickness skin defect wound model for the evaluation of the influence exerted by inflammatory factors on the healing of wounds *in vivo*. Mice were randomly divided into P311 WT and P311 siRNA groups, each group containing six subgroups: control, solvent, IL-1β 200 ng, IL-1βab 2 μg, TNF-α 200 ng, and TNF-αab 2 μg groups (10 mice in each subgroup, a total of 120 mice). In brief, using 1% pentobarbital, BALB/c mice received intraperitoneal anesthesia. We trimmed dorsal fur and washed the skin with 75% alcohol. We created 6-mm diameter full-thickness wounds using a punch on each side of the back. To represent the initial wound area, we placed a 6-mm-diameter sterile round marker next to each wound, and then used a digital camera to obtain images of the wounds immediately. Subsequently, onto the gauze surface, we glued a piece of biological membrane (NPWT-1, Negative Pressure Wound Therapy Kit, China). We obtained images of the wounds at days 3 and 7 post-surgery. The wound healing rate and complete healing time were compared between the two groups. Using IPP 6.0 software, we measured wound areas. We calculated wound healing rate as follows:

Wound healing rate% = (the original wound area – the remaining wound area)/original wound area × 100%.

### Hematoxylin-Eosin (H&E) Staining and Histological Analysis

At day 7 after surgery, the mice were sacrificed. We acquired wound tissue samples, treated them using 4% paraformaldehyde and immersed them in paraffin. Using traditional approaches, we performed HE staining. Using the Image J 1.48V software (NIH, United States), the neonatal epithelial length, granulation tissue thickness, and number of blood vessels were photographed and counted.

### Immunohistochemical Staining

Using immunohistochemistry, we measured the expression of CD31, vascular endothelial growth factor (VEGF), proliferating cell nuclear antigen (PCNA), and P311 in wound tissues sections at day 5 post-surgery. In brief, wound tissue sections underwent deparaffinization and rehydration, and then they were incubated at 95°C in a sodium citrate buffer bath to retrieve the heat-mediated antigen. We then stored the sections in 3% H_2_O_2_ for 15 min at 25°C. Next, we cleaned the sections using PBS three times, subsequently blocked them in 10% normal goat serum (Zhongshan Biology Company) for 30 min at 37°C, and then incubated them using the primary antibody at 4°C overnight. Subsequently, we washed the sections with PBS three times, and incubated with the biotinylated goat anti-rabbit IgG antibody (Zhongshan Biology Company) for 30 min at 37°C. Next, we stored the sections in the avidin–peroxidase reagent (Zhongshan Biology Company) at 37°C for 30 min. Finally, we stained the sections by hematoxylin and 3,3′-diaminobenzidine tetrahydrochloride, and then obtained images using an optical microscope (LEICA, Germany, CTR6000). The primary antibodies were as follows: PCNA (1:200, Abcam, ab15497); P311 (1:100, Novus, NBP1-84315); VEGF (1:100, Abcam, ab46154); and CD31 (1:100, Abcam, ab28364).

### Statistical Analysis

Using two-way analysis of variance (ANOVA) (for more than two groups) and one-way ANOVA (for two groups) based on the Origin software, we analyzed significant differences between groups. The experimental data were expressed as mean ± standard. *p* < 0.05 was considered statistically significant. The percentages reported for the various factors studied are devoid of standard deviation.

## Results

### Influences Exerted by TNF-α and IL-1β on the Proliferation and Cytoskeleton of Vascular Endothelial Cells

**FIGURE 1:**
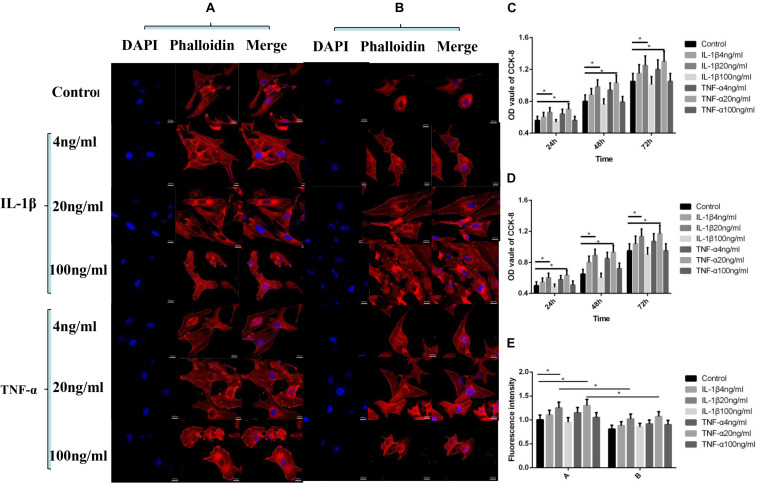
Effects of IL-1β and TNF-α on proliferation and cytoskeleton of vascular endothelial cells. Mean ± standard deviation (SD) denote the experimental results; * indicates *P* < 0.05. **(A)** Samples without P311 siRNA intervention. **(B)** Samples with P311 siRNA intervention. **(C)** Statistical cell proliferation results of **(A)**. **(D)** Statistical cell proliferation results of **(B)**. **(E)** Representative cell skeleton staining images of **(A)** and **(B)**.

As shown in Figure 1, according to the 24–72 h nuclear staining (DAPI) results, cell proliferation activity was the greatest when IL-1β and TNF-α were at 20 ng/ml concentration (*P* < 0.05, Figures 1A,C), and the addition of P311 siRNA did not have a significant effect on the trend of cell proliferation (*P* < 0.05, Figures 1B,D). Based on the 24-h cytoskeletal staining (phalloidin) results, the cytoskeletal protein activity was the greatest when both TNF-α and IL-1β were 20 ng/ml (*P* < 0.05, Figure 1E).

### Influences Exerted by TNF-α and IL-1β on the Migration of Vascular Endothelial Cells

**FIGURE 2:**
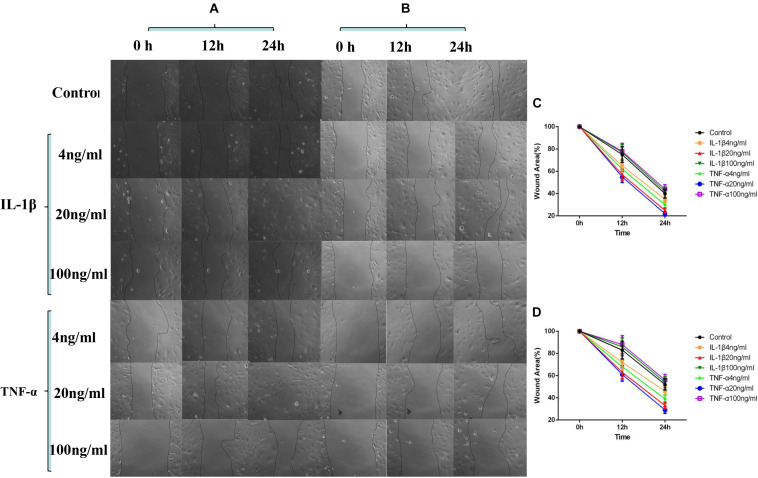
Effects of IL-1β and TNF-α on migration of vascular endothelial cells. The experimental results are expressed as mean ± standard deviation (SD); * indicates *P* < 0.05. **(A)** Samples without P311 siRNA intervention. **(B)** Samples with P311 siRNA intervention. **(C)** Statistical cell migration rate of **(A)**. **(D)** Statistical cell migration rate of **(B)**.

As shown in Figure 2, by comparing the cell migration rate at 24 h, we observed that a percentage of the wound area decreased the most when TNF-α and IL-1β were at 20 ng/ml concentration, suggesting that the cell migration rate was the fastest at this concentration (*P* < 0.05, Figures 2A,C). The addition of P311 siRNA did not have a significant influence on the trend of cell migration (*P* < 0.05, Figures 2B,D). However, cell migration was inhibited when compared with the group without the addition of P311 siRNA under the same stimulation conditions (*P* < 0.05, Figures 2B,D).

### Influences Exerted by TNF-α and IL-1β on Vascular Endothelial Cell Angiogenesis

**FIGURE 3:**
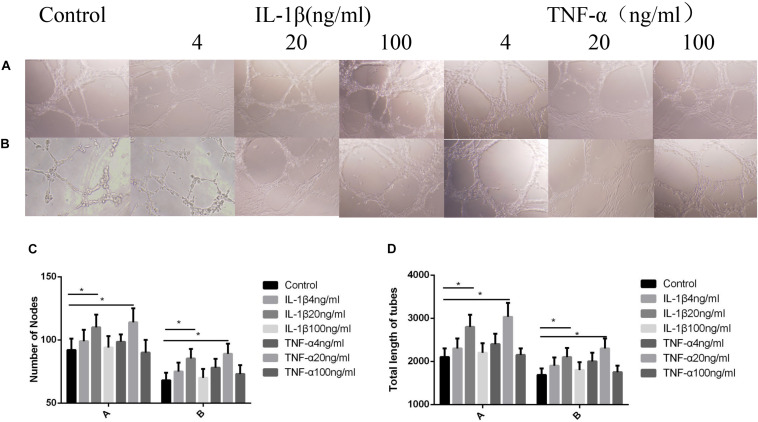
Influences exerted by IL-1β and TNF-α on vascular endothelial cell angiogenesis. Mean ± standard deviation (SD) denote the experimental results; * indicates *P* < 0.05. **(A)** Samples without P311 siRNA intervention. **(B)** Samples with P311 siRNA intervention. **(C)** Number of blood vessels corresponding to **(A)** and **(B)**. **(D)** Length of blood vessels in correspondence to **(A)** and **(B)**.

As shown in Figure 3, when the TNF-α and IL-1β concentrations were 20 ng/ml, the number of blood vessels was at its highest (*P* < 0.05, Figures 3A,C), and the length of the blood vessels were at their longest (*P* < 0.05, Figures 3A,D). After the addition of P311 siRNA, the number and length of blood vessels decreased, whereas blood vessels number and length at 20 ng/ml remained the highest (*P* < 0.05, Figures 3B–D).

### Influences Exerted by TNF-α and IL-1β on NF-κB Phosphorylation and P311 mRNA Expression

**FIGURE 4:**
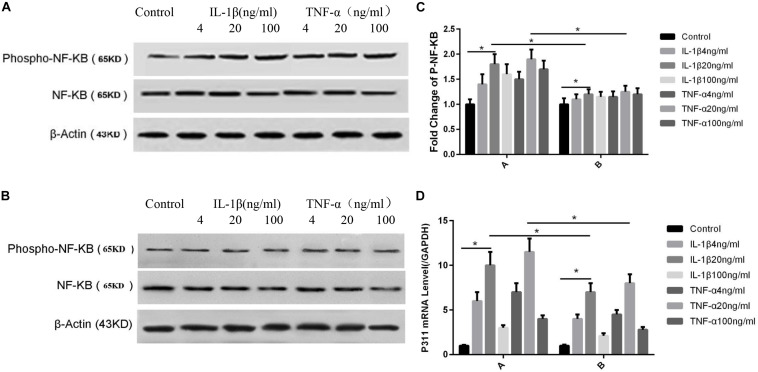
Influences exerted by IL-1β and TNF-α on NF-κB phosphorylation and P311 mRNA expression. Mean ± standard deviation (SD) denote the experimental results; * indicates *P* < 0.05. **(A)** Samples without P311 siRNA intervention. **(B)** Samples with P311 siRNA intervention. **(C)** Corresponding NF-κB phosphorylation level changes in **(A)** and **(B)**. **(D) A** indicated P311 mRNA level without the NF-κB inhibitor CAPE and **B** indicated P311 mRNA level when adding the NF-κB inhibitor CAPE.

As shown in Figure 4, the NF-κB phosphorylation levels were highest when IL-1β and TNF-α were at 20 ng/ml concentration (*P* < 0.05, Figures 4A,C-A). Phosphorylation levels were lower after the addition of P311 siRNA; however, the levels were still maximal when TNF-α and IL-1β levels were at 20 ng/ml (*P* < 0.05, Figures 4B,C-B). Regarding the effect of various IL-1β and TNF-α concentrations on the expression of P311 mRNA, the expression level of P311 mRNA was found to be the highest when the TNF-α and IL-1β levels were at 20 ng/ml (*P* < 0.05, Figure 4D-A). The expression levels of P311 mRNA decreased after the addition of the NF-κB inhibitor CAPE; however, their expression levels remained the highest at 20 ng/ml of IL-1β and TNF-α stimulation (*P* < 0.05, Figure 4D-B).

### Effects of NF-κB on the Activity of the P311 Promoter in Vascular Endothelial Cells and Analysis of Binding Sites

**FIGURE 5:**
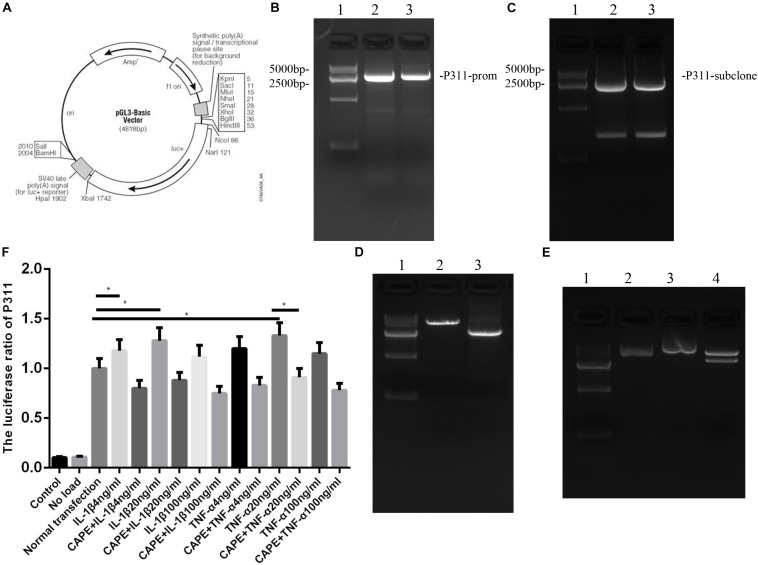
Influences exerted by NF-κB on the activity of the P311 promoter in vascular endothelial cells and analysis of binding sites. Mean ± standard deviation (SD) denote the experimental results; * indicates *P* < 0.05. **(A)** P311 promoter expression plasmid. **(B)** P311 electropherogram. **(C)** P311 subclone band. **(D)** Luciferase activity report under CAPE intervention. **(E)** Luciferase activity report. **(F)** The Iuciferase ratio of P311.

As shown in Figure 5, to measure the effect of NF-κB on the activity of the P311 promoter, the NF-κB adenovirus and P311 promoter region dual luciferase reporter gene vector, namely, the P311 promoter expression plasmid (Figure 5A) was constructed, and total protein was extracted and luciferase activity was measured. The expression levels of P311 in the normal transfection group were significantly upregulated (*P* < 0.05). P311 expression levels were also upregulated when stimulated with 20 ng/ml of IL-1β and TNF-α (*P* < 0.05). However, P311 expression levels decreased significantly when the NF-κB phosphorylation inhibitor CAPE was added.

### Effects of Different Stimulus Factors on Mice Wound Healing

**FIGURE 6:**
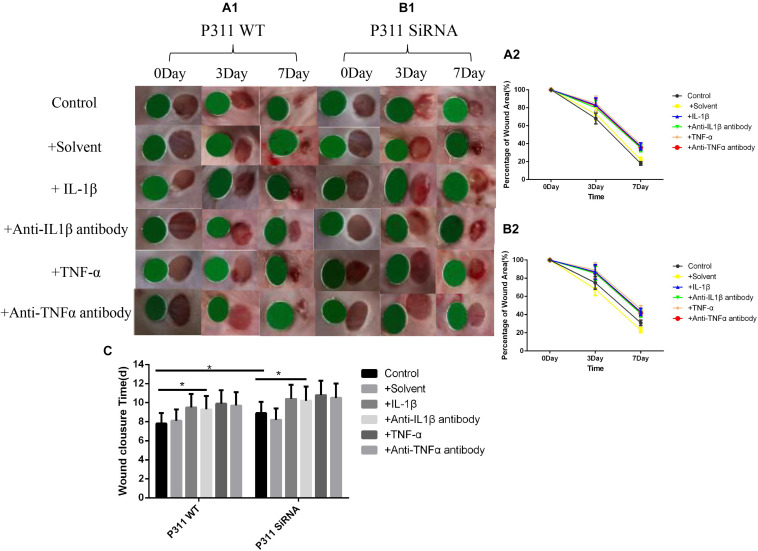
Effects of various stimulus factors on wound healing. Mean ± standard deviation (SD) denote the experimental results; * indicates *P* < 0.05. **(A1)** P311 WT mice wound changes. **(B1)** P311 siRNA wound changes. **(A2)** Corresponding wound changes data of **(A1)**. **(B2)** Corresponding wound changes data of **(B1)**. **(C)** Complete wound healing times of **(A1)** and **(B1)** groups.

As shown in Figure 6, on the seventh day post-surgery, the percentage of the remaining wound area was 18% in the control group, and 37, 35, 39, and 36% in the IL-1β, IL-1βab, TNF-α, and TNF-αab groups, respectively, suggesting that either sharply increasing or reducing the inflammatory stimulus caused delayed wound healing (*P* < 0.05). Regarding the control group with P311 siRNA treatment, the percentage of the remaining wound area was 31%, suggesting that P311 siRNA significantly delayed wound healing (*P* < 0.05). It is worth noting that, when the above-mentioned inflammatory stimulus factors were combined with P311 siRNA, the wound healing rate delay of the two combined factors was significantly lower than the sum of the wound healing rate delays of the two individual factors, suggesting that the effect of P311 on wound healing may involve the NF-κB regulatory mechanism.

### Effect of Different Stimulus Factors on Neonatal Epithelial Length, Number of Blood Vessels, and Granulation Tissue Thickness

**FIGURE 7:**
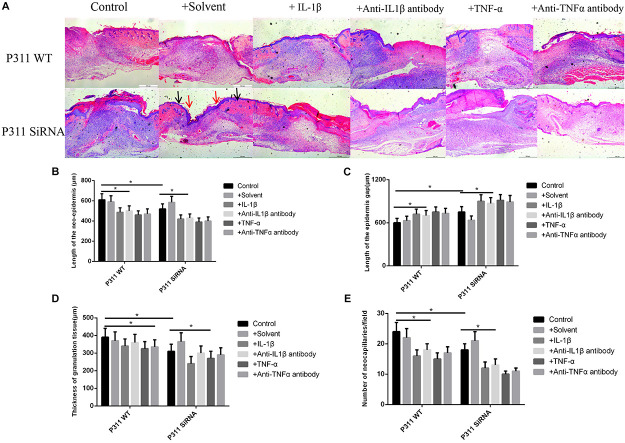
Effect of various stimulus factors on neonatal epithelial length, granulation tissue thickness, and number of blood vessels. Mean ± standard deviation (SD) denote the experimental results; * indicates *P* < 0.05. **(A)** Representative histological images of the length of neonatal epithelium under different stimulus factors in P311 WT and P311 siRNA groups. **(B)** Neonatal epithelium length measurement. **(C)** Epidermis gap length measurement. **(D)** Measurement of granulation tissue thickness. **(E)** Measurement of the number of neocapillaries.

As shown in Figure 7, the neonatal epithelial length (B), the number of blood vessels (E), and the granulation tissue thickness (D) of the IL-1β, IL-1βab, TNF-α, and TNF-αab groups were smaller than those of the control group, suggesting that either sharply increasing or reducing inflammatory stimuli can affect neonatal epithelial length, granulation thickness, and angiogenesis (*P* < 0.05). The neonatal epithelial length, granulation tissue thickness, and number of blood vessels in the control group with P311 siRNA treatment were also smaller than those of the control group, suggesting that P311 siRNA significantly affected these indicators, thereby affecting wound healing (*P* < 0.05).

### Effects of Different Stimulus Factors on the Expression of P311 in Wound Tissues

**FIGURE 8:**
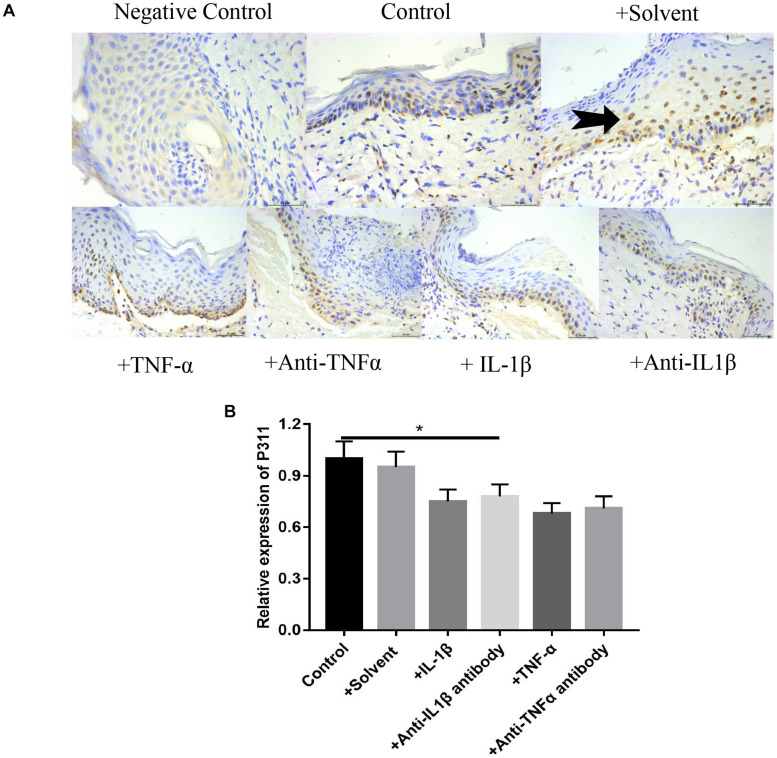
Effects of various stimulus factors on the expression of P311 in wound tissues. Mean ± standard deviation (SD) denote the experimental results; * indicates *P* < 0.05. **(A)** Representative IHC stains for P311 in wound tissues under different stimulus factors. **(B)** Quantitation of the P311 levels in **(A)**.

As shown in Figure 8, based on the immunohistochemical staining of P311 on the 7-day wound specimen, we found that the relative expression levels of P311 were control > solvent group > IL-1βab > IL-1β > TNF-αab > TNF-α, suggesting that either sharply increasing or reducing inflammatory stimulus reduced expression levels of P311 in wound tissues (*P* < 0.05).

### Effect of Different Stimulus Factors on the Expression of PCNA, CD31, and VEGF in Wound Tissues

**FIGURE 9:**
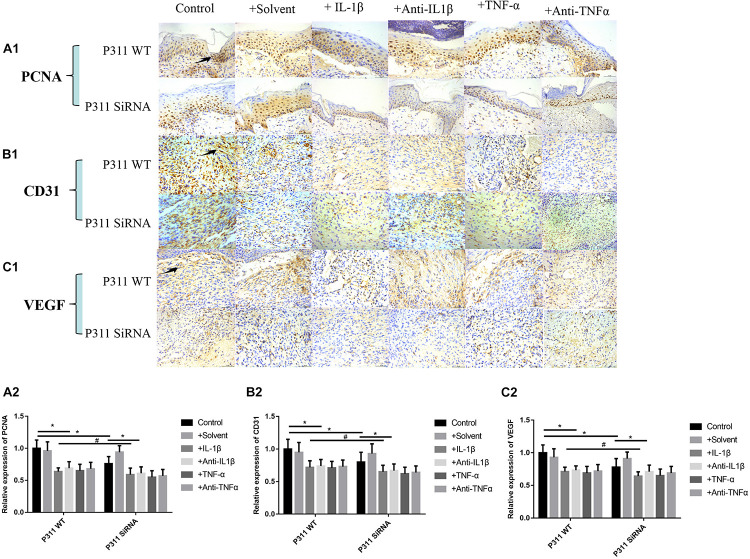
Effect of different stimulus factors on the expression of PCNA, CD31, and VEGF in wound tissues. Mean ± standard deviation (SD) denote the experimental results; * indicates *P* < 0.05, ^#^ indicates *P* > 0.05. **(A1)** Representative IHC stains for PCNA in wound tissues under different stimulus factors. **(B1)** Representative IHC stains for CD31 in wound tissues under different stimulus factors. **(C1)** Representative IHC stains for VEGF in wound tissues under different stimulus factors. **(A2)** Quantitation of the PCNA levels in **(A1)**. **(B2)** Quantitation of the CD31 levels in **(B1)**. **(C2)** Quantitation of the VEGF levels in **(C1)**.

As shown in Figure 9, relative expression levels of PCNA (Figures 9A1,A2), CD31 (Figures 9B1,B2), and VEGF (Figures 9C1,C2) in IL-1β, IL-1βab, TNF-α, and TNF-αab groups were significantly lower than those of the control group (*P* < 0.05), suggesting that either a dramatic increase or decrease of inflammatory stimulus factors can affect expression levels of these proteins in wounds. In addition, relative expression levels of PCNA, CD31, and VEGF in the control group with P311 siRNA treatment were also lower than those of the control group, suggesting that P311 siRNA affected wound healing by influencing these factors (*P* < 0.05).

## Discussion

As shown in Figure 1, the cell proliferation and cytoskeletal protein activity was the greatest when TNF-α and IL-1β were at 20 ng/ml (*P* < 0.05). As shown in Figures 2, 3, when TNF-α and IL-1β concentrations were 20 ng/ml, comparing the cell migration rates at 24 h, the number and length of blood vessels were at their greatest (*P* < 0.05). These findings suggest that P311 facilitates regeneration of nerves and lungs, as well as glioma invasion, and induces myofibroblast differentiation (Bossi and Lehner, 2009; DeCicco-Skinner et al., 2014) and cell migration (Fujitani et al., 2004; Herkenne et al., 2015). After the addition of P311 siRNA, the number and length of blood vessels decreased (*P* < 0.05). This suggests that angiogenesis is closely related to P311.

P311 knockout mice exhibited vascular hypotension, hypotonic blood vessels, and downregulated vascular smooth muscle cell contractility in comparison with the wild-type (Johnson and Wilgus, 2014), as well as variable behavior responses in memory and learning. Nevertheless, the biological molecular mechanisms of P311 remain unclear. The PEST domain (abound with Pro, Glu, Ser, and Thr) in the N-terminus of P311 is considered a functional motif that probably serves as a mechanism indicating function, because the domain has been found in ephemeral proteins as well (e.g., signal molecules, cytokines, and transcription factors) (Adams and Alitalo, 2007; Koch and Claesson-Welsh, 2012). Recently, P311 has been found to upregulate the activity of Rac1 and Rho A (Carmeliet, 2005), i.e., vital signal transducers to form blebs, invadopodia, filopodia, and lamellipodia during cell migration. P311 was found to induce EpMyT $ (epidermal stem cell transdifferentiating into myofibroblasts) via TGFb1/Smad signaling.

After stimulating rat astrocytes and chondrocytes, IL-1β activated NF-κB B through the traf6-tak-ikk pathway, and NF-κB inhibitors blocked the effects of IL-1β (18). Other studies demonstrated that TNF-α combined with the human VECs TNFR1/TNFR2 also activated NF-κB B through the traf2-ikk pathway to promote the production of vascular cell adhesion factor 1, thereby promoting vascular repair and regeneration (Cha et al., 2005). Scortegagna et al. found that high expression levels of HIF1 alpha in mouse keratinocytes caused excessive phosphorylation of kappa B and phosphorylation of P65 ser276, thereby increasing the activity of NF-κB B. According to our own preliminary experiments, IL-1β, TNF-α, and hypoxic stimulation all caused increased P311 expression, and several studies confirmed that all three can play biological roles through NF-κB, suggesting that NF-κB is likely to be vital to P311 expression after skin injury. Our preliminary studies also found that anoxia, IL-1β, and TNF-α promote the phosphorylation of the VEC NF-κB B. As shown in Figure 4, NF-κB phosphorylation levels were found to be the highest when IL-1β and TNF-α were at 20 ng/ml (*P* < 0.05, Figures 4A,C-A). Phosphorylation levels decreased after the addition of P311 siRNA; however, the levels remained maximal when TNF-α and IL-1β were at 20 ng/ml (*P* < 0.05, Figures 4B,C-B). Regarding the effects of various TNF-α and IL-1β concentrations on expression levels of P311 mRNA, expression levels of P311 mRNA were found to be maximal when TNF-α and IL-1β were at 20 ng/ml (*P* < 0.05, Figure 4D-A). Expression levels of P311 mRNA decreased after the addition of the NF-κB inhibitor CAPE; however, its expression levels remained the highest at 20 ng/ml of IL-1β and TNF-α stimulation (*P* < 0.05, Figure 4D-B).

NF-κB B is a highly conserved nuclear transcription factor widely found in mammalian cells. Studies suggested that NF-κB B is vital to immune and inflammatory processes and is involved in the regulation of the expression of various protein molecules within the cell. Therefore, NF-κB B may be an intermediate molecule involved in the inflammatory stimulation and initiation of the expression of specific proteins. At rest, NF-κB combined with the inhibitor of NF-κB B (I) has no function. When the cells are stimulated by an upstream ligand (such as IL-1β and TNF-α), the proteins of kappa B degrade under the action of IKK and the release NF-κB. The selective activation of the transcription of downstream genes after specific stimulation remains one of the most important scientific problems in the field of NF-κB. According to our recent bioinformatics prediction, we found that there were three possible binding sites of NF-κB B in the promoter of P311 (see the basis of work for details), further suggesting that IL-1, TNF-α, and hypoxia in the wound promote the expression of P311 through the NF-κB signal pathway after skin injury. As shown in Figure 5, to measure the effect of NF-κB on the activity of the P311 promoter, we constructed an NF-κB adenovirus and P311 promoter region dual luciferase reporter gene vector, namely, the P311 promoter expression plasmid (Figure 5A), total protein was extracted, and luciferase activity was detected. The expression levels of P311 in the normal transfection group were significantly upregulated (*P* < 0.05). P311 expression levels were also greater when stimulated with 20 ng/ml concentration of IL-1β and TNF-α (*P* < 0.05). However, the expression levels of P311 decreased significantly when the NF-κB phosphorylation inhibitor CAPE was added.

To determine the effect of P311 on angiogenesis in wound healing, a full-thickness excisional skin wound model was created with P311 KO and P311 WT mice. In macroscopic analysis, wound healing was significantly delayed in P311 KO mice. Moreover, the average wound closure time was even longer in P311 KO mice. Wounds from P311 KO mice displayed thinner granulation tissue and CD31 + EC numbers were reduced in granulation tissue in P311 KO mice, which was further confirmed using WB. The results suggest that P311 promotes wound healing by enhancing angiogenesis in granulation tissue. Taken together, the data suggest that P311 regulates angiogenesis in granulation tissue by directly altering endothelial cell response. As shown in Figure 6, on the seventh day post-surgery, the percentage of the remaining wound area was 18% in the control group, and was 37, 35, 39, and 36% in the IL-1β, IL-1βab, TNF-α, and TNF-αab groups, respectively, suggesting that either sharply increasing or reducing the inflammatory stimuli caused delayed wound healing (*P* < 0.05). Regarding the control group with P311 siRNA treatment, the percentage of the remaining wound area was 31%, suggesting that P311 siRNA significantly delayed wound healing (*P* < 0.05). It is worth noting that when these inflammatory stimulus factors were combined with P311 siRNA, the wound healing rate delay of the two combined factors was significantly lower than the sum of the wound healing rate delays of the two individual factors, suggesting that the effect of P311 to promote wound healing may be included in the NF-κB regulatory mechanism.

Here, for the first time, we demonstrated that P311 promoted angiogenesis in wound healing by altering the endothelial response. This function was supported by the following findings of our present study: (1) P311 colocalized with vWF + ECs in the murine dermis, and dermal microvascular endothelial cells (mDMECs) expressed protein P311 and highly expressed it under injury conditions; (2) P311-deficient mDMECs showed decreased tube formation and migration *in vitro*; (3) *in vivo*, compared with the P311 WT mice, a significantly lower number of ECs was detected in subcutaneous Matrigel implants in P311 KO mice; and (4) P311 KO mice showed impaired granulation tissue formation and fewer CD31 + ECs in the granulation tissues in wound healing. As shown in Figure 7, the neonatal epithelial length (B), the granulation tissue thickness (D), and the number of blood vessels (E) of the IL-1β, IL-1βab, TNF-α, and TNF-αab groups were smaller than those of the control group, suggesting that either sharply increasing or reducing the inflammatory stimuli affects neonatal epithelial length, granulation thickness, and angiogenesis (*P* < 0.05). The neonatal epithelial length, granulation tissue thickness, and number of blood vessels in the control group with P311 siRNA treatment were also smaller than that of the control group, suggesting that P311 siRNA significantly alters these indicators, thereby affecting wound healing (*P* < 0.05).

Microvascular endothelial cells are the primary parenchymal cell participating in wound angiogenesis. Nevertheless, experiments are rarely conducted to determine whether microvascular endothelial cells are likely to express P311. With the use of immunofluorescence, we found that P311 colocalized with vWF + ECs in the murine dermis. Furthermore, vWF and P311 expressions were detected in isolated mDMECs at steady state and injury conditions. These findings suggest that mDMECs expressed P311 and highly expressed it *in vivo* and *in vitro* under injury conditions. Other studies suggested that cells from normal tissue had low P311 expression that was significantly upregulated in cells from injured tissue; this suggests that P311 is likely to be an injury-dependent protein (Cheng et al., 2017). Subsequently, with the use of the isolated mDMECs, the effect of P311 deficiency on EC function was determined *in vitro*. According to previous reports, P311-deficient mDMECs exhibited reduced migration ability. Tube formation in P311-deficient mDMECs was reduced as well. Nevertheless, the cell proliferation was basically identical in both mDMECs. In the subcutaneous Matrigel implant, neoangiogenesis was significantly lower in P311 KO mice. This suggests that P311 might reduce neoangiogenesis by altering endothelial cell responses. As shown in Figure 8, based on the immunohistochemistry staining of P311 in 7-day wound specimens, we found that relative expression levels of P311 were control > solvent group > IL-1βab > IL-1β > TNF-αab > TNF-α, suggesting that either sharply increasing or reducing inflammatory stimulus reduced expression levels of P311 in wound tissues (*P* < 0.05). In P311 KO mice wounds, decreased VEGF and TGFb1 expression levels were also found. These two are vital angiogenic factors modifying angiogenesis and vasculogenesis. By binding to the tyrosine kinase receptor VEGFR2, VEGF regulates angiogenesis, which induces dimerization, receptor phosphorylation, and downstream signaling pathways (Adams and Alitalo, 2007; Koch and Claesson-Welsh, 2012). As endothelial cells can secrete VEGF and express P311, it is inferred that P311 is likely to cause VEGF to exert stimulating effects, thereby altering endothelial cell responses and subsequently altering angiogenesis. The P311-VEGF/VEGFR2-ECs response is likely to be a mechanism of angiogenesis. Further study should test this hypothesis. TGF-b signaling pathways have been demonstrated to be vital factors for vasculogenesis and angiogenesis through several genetic manipulations of the pathway components. In recent years, the Schuger group and our own group found that P311 was a regulator of TGF-bs and was an RNA-binding protein for the stimulation of TGF-bs translation *in vitro* and *in vivo* (Cheng et al., 2017). These findings suggest that, in wound healing, P311 is likely to regulate angiogenesis by modulating TGFb secretion. Furthermore, TGF-b was found to facilitate VEGF secretion. These findings suggest that P311 is likely to regulate angiogenesis via the P311-TGFb-VEGF/VEGFR2 signaling pathway. The confirmation of the exact mechanism by which P311 regulates angiogenesis in wound healing will require more studies. As shown in Figure 9, relative expression levels of PCNA (Figures 9A1,A2), CD31 (Figures 9B1,B2), and VEGF (Figures 9C1,C2) in the IL-1β, IL-1βab, TNF-α, and TNF-αab groups were remarkably lower than those of the control group (*P* < 0.05), suggesting that either a dramatic increase or decrease of inflammatory stimulus factors regulates the expression of these proteins in wounds. The relative expression levels of PCNA, CD31, and VEGF in the control group with P311 siRNA treatment were also lower than those of the control group, suggesting that P311 siRNA affected wound healing by influencing expression of these factors (*P* < 0.05).

In conclusion, we demonstrated for the first time that p311 promotes angiogenesis during wound healing by altering endothelial responses.

## Data Availability Statement

All datasets presented in this study are included in the article/supplementary material.

## Ethics Statement

The Southwestern Hospital Institutional Review Board reviewed and approved all protocols involving animals.

## Author Contributions

All authors made contribution to designing this study. DZ and GL designed and performed the experiments. TL, WQ, and SW were responsible for data analysis and for producing figures. DZ and WH wrote the manuscript. GL was responsible for obtaining funds. All authors contributed to interpreting results and revising the manuscript.

## Conflict of Interest

The authors declare that the research was conducted in the absence of any commercial or financial relationships that could be construed as a potential conflict of interest.
